# Associations between HIV Antiretroviral Therapy and the Prevalence and Incidence of Pregnancy in Rakai, Uganda

**DOI:** 10.1155/2011/519492

**Published:** 2011-01-19

**Authors:** Fredrick. E. Makumbi, Gertrude Nakigozi, Steven. J. Reynolds, Anthony Ndyanabo, Tom Lutalo, David Serwada, Fred Nalugoda, Maria Wawer, Ron Gray

**Affiliations:** ^1^School of Public Health, Makerere University, 7072 Kampala, Uganda; ^2^Rakai Health Sciences Program, 279 Kalisizo, Uganda; ^3^National Institute of Allergy and Infectious Diseases, National Institutes of Health, Bethesda, MD 20892, USA; ^4^School of Public Health, Johns Hopkins Bloomberg, MD 21205, USA

## Abstract

*Background*. Use of antiretroviral therapy (ART) may be associated with higher pregnancy rates. *Methods*. The prevalence and incidence of pregnancy was assessed in 712 HIV+ pre-ART women of reproductive age (WRA) (15–45) and 244 HIV+ WRA initiating ART. Prevalence rate ratios (PRR), incidence rate ratios (IRR), and 95% confidence interval (CI) were assessed. *Results*. The incidence of pregnancy was 13.1/100 py among women in pre-ART care compared to 24.6/100 py among women on ART (IRR = 0.54; 95% CI 0.37, 0.81, *p* < 0.0017). The prevalence of pregnancy at ART initiation was 12.0% with CD4 counts 100–250 compared with 3.2% with CD4 <100 (PRR = 3.24, CI 1.51–6.93), and the incidence of pregnancy while on ART was highest in women with a good immunologic response. Desire for more children was a very important factor in fertility. *Conclusion*. ART was associated with increased pregnancy rates in HIV+ women, particularly those with higher CD4 counts and good immunologic response to therapy, suggesting a need to strengthen reproductive health services for both women and their partners that could address their fertility decisions/intentions particularly after ART initiation.

## 1. Introduction

The HIV/AIDs epidemic remains a serious public health challenge, as well as a social dilemma especially among women of childbearing age [1] who were 46% of the HIV global burden [2]. HIV infected women face difficult decisions regarding childbearing. HIV-infection compromises their immunity which further aggravates their chances of conception as well as sustaining a pregnancy to term. Reduced fertility among HIV-positive women has been reported in sub-Saharan Africa [3–7]. Fertility is not impaired during early HIV infection [8, 9] but declines with disease progression, and the reduction is greatest with onset of AIDS [10]. The mechanisms through which fertility rates are reduced by HIV are not fully understood, but higher viral load and decreased CD4 counts with advanced HIV disease are likely to be implicated [11]. Also, progression to AIDS leads to decreased general health and well-being that may be associated with reduced sexual activity. However, the advent of ART has improved the general health and may increase sexual activity, and improved survival prospects may increase the desire for more children [12–15]. Desire for more children may also be due to improvements in pregnancy care that have reduced the risk of vertical transmission, especially if pregnant women receive timely ART or if they deliver by caesarean section and avoid breastfeeding when recommended. However, even in the era of HIV/AIDS other predictors of fertility including age, marital status, level of education, and socioeconomic status still play a significant role in determining fertility [16–20]. A combination of all these factors may be associated with the currently observed increase in pregnancy rates among women on ART [21, 22]. Although HIV-infected women's fertility desires have been studied, there is limited information on the possible role of the improving immunological and virological outcomes on the actual fertility among women initiated on ART. Understanding the effects of ART on women's fertility is critically important for health service providers to be able to protect the reproductive rights of HIV-infected women through creating increased access to sexual and reproductive health services and sexuality education, so that women may decide freely and responsibly on their family size through spacing and timing of their pregnancies [23] In this paper we therefore assess the association between HIV infection, ART use, and pregnancy rates, taking into account immunological and virological status, in a community-based HIV care program in Rakai district, Uganda.

## 2. Methods

### 2.1. Study Setting, Population, and HIV Treatment and Care

Since June 2004, with funding from the President's Emergency Plan For AIDS Relief (PEPFAR), the Rakai Health Sciences Program (RHSP) in Uganda has offered HIV care and ART free of charge. HIV care, offered to all HIV-infected persons, includes cotrimoxazole prophylaxis, treatment for opportunistic infections, reproductive health services, treatment of opportunistic infections, cotrimoxazole prophylaxis, provision of a basic care package, health education, and HIV counseling. Individuals are initiated on ART at a CD4 cell count ≤250 cells/mm^3^ or WHO Stage IV disease. General HIV care and ART is provided via 17 mobile community-based out-patient clinics, called Suubi or “Hope” clinics. All patients are offered ongoing HIV counseling, health education including family planning, prevention of mother to child transmission of HIV, and counseling on reduction of risky sexual behaviors for example, multiple sexual partners. At each visit, general health status is evaluated, and use of family planning methods are ascertained. 

At the time data were collected for these analyses, individuals on HIV care but not yet started on ART were seen every 3 to 6 months depending on CD4 cell count. Patients initiating ART were followed weekly for the first month, biweekly for the subsequent 2 months, monthly until one year on treatment, and then every 2 months thereafter. CD4 counts were assessed every 3 or 6 months prior to ART, at time of ART initiation, and every 3 months thereafter. Viral load was tested at time of ART initiation and then at 6 monthly intervals. Two weeks prior to ART initiation, women were interviewed regarding their and their sexual partner's fertility desires and intentions; the interview was repeated at 6 months and annually thereafter. Women provided written informed consent for interview, and were part of the ongoing Rakai community cohort surveillance (RCCS). The RCCS study was approved by the Science and and Ethics Committee of the Uganda Virus Research Institute, the Ugandan National Council for Science and Technology, and the Western Institutional Review Board.

### 2.2. Definition of Key Variables

Pregnancy was detected through self-report and hCG urine test for those uncertain of their pregnancy status or whose last normal menstrual period (LNMP) was more than 30 days prior to interview, with the exception of women using Depo-Provera or norplant, and those who were postmenopausal or had lactational amenorrhoea <2 months, who were excluded from this analysis. Prevalent pregnancies were defined as those detected at or within two-weeks of enrolment into HIV care or ART initiation. Incident pregnancies were defined as those first detected more than 2 weeks after entry in HIV care or ART initiation. (The two-week window was included because, on average, using the pregnancy kits available to this program, hcg could only be detected in a pregnant woman's urine after at least 10 days.) If a woman had more than one pregnancy during follow-up, only the first pregnancy was considered for the incidence analysis, and subsequent observation time was censored.

All women initiating ART had CD4 counts of ≤250 cells/mm^3^ and were categorized into CD4 counts <100 or 100–250 cells/mm^3^. Desire for more children was categorized as both partners wanting a child, only the male or the female partner wanting a child, or neither wanting a child.

 We also grouped responses to desire for more children into four categories as (i) *both partners did not *want a child if neither wanted more children, (ii) *only male partner* wanted *a child,* (iii) o*nly the woman* wanted more children, (iv) *both wanted (more) children*.

### 2.3. Statistical Analysis

The two key outcomes for this analysis were prevalent pregnancy defined as a pregnancy at enrolment into HIV care for the pre-ART period, or at ART initiation or within the first 2 week of follow-up for those initiated on ART. Incident pregnancy was defined as the first pregnancy during subsequent visits starting at the 2nd week followup following ART initiation and at 3 or 6 months visits after enrolment in HIV care prior to ART initiation, among women who were not pregnant at time of enrollment in care or initiation of ART.

#### 2.3.1. Analyses of Incident Pregnancy during HIV Care prior to ART and during ART

 The prevalence of pregnancy at two time points (at the time of HIV care initiation and at ART initiation) was determined as the number of women pregnant at time of HIV care or ART initiation divided by the total number of sexually active women of childbearing age (15–45 years) starting HIV care or ART (premenarche women and women using depo-provera or norplant, and those who were post menopausal or had lactational amenorrhoea <2 months were excluded from analysis). Prevalence risk ratios (PRR) and 95% confidence intervals (CI) were estimated using generalized linear models (glm) with family of binomial and log link. Models for adjusted PRR included baseline CD4 and other factors statistically significant at *p* < 0.15 in univariate analyses or potential confounders.

 The incidence of new pregnancies detected between the second week up to the 48th week during two time periods (after initiation of HIV care and after initiation of ART) was estimated as the number of first pregnancies in each time period divided by the total person-years (py) accrued in that time period before pregnancy detection or before censoring at week 48. Incidence rate ratios (IRR) of first pregnancy and 95% confidence intervals (CI) were estimated using Poisson regression with observation time as an offset. 

Covariates assessed in the pre-ART period pregnancy incidence included CD4 at screening for enrolment into HIV care, categorized as 251–350 and 351+ cells/mm^3^, age at enrolment, marital status, family planning use, and breastfeeding in the past 12 months. In multivariable models, we adjusted for factors statistically significant at *p* < 0.15 or potential confounders. The main factor of interest in this analysis was CD4 counts at enrolment into HIV care.

For the ART treatment period, covariates included change in CD4 counts between baseline (the time of ART initiation) and week 12 of treatment, divided into three categories: baseline and week 12 CD4 were both <100 cells/mm^3^, baseline CD4 was <100, week 12 CD4 was 100+ cells/mm^3^ and both baseline CD4 and week 12 were 100+ cells/mm^3^, age at ART initiation, marital status, baseline desire for children, body mass index (BMI) calculated as (Weight in Kilograms/(Height in Meters) × (Height in Meters)) at week 12, family planning use at baseline, and HIV viral load at week 24. In multivariable models, we adjusted for factors which were statistically significant at *p* < 0.15 in univariate analyses or potential confounders. Using Kaplan-Meier survival curves, we also assessed the association between CD4 counts and the cumulative probabilities of incident pregnancies comparing women in the three categories of change in CD4 between baseline and week 12. The log-rank test was used to assess differences in the cumulative probabilities of pregnancy between these CD4 change categories.

In both, the generalized linear models (glm), for estimation of the PRR and IRR, we adjusted for clustering of observation at the mobile clinic (hub) levels, because women attending specific mobile clinics were more likely to be similar in various characteristics than those attending others.

## 3. Results

Table 1 shows the characteristics of women who enrolled into pre-ART HIV care in the RHSP. The majority were aged 25–34 years (54.8%), currently married (48.2%), and those with no sexual partners in the past 6 months (64.4%). About 40% had parity of 4 or more children, and 37% were prime gravidae. About 18.4% used medications to prevent pregnancies, but injectables were the most commonly reported pregnancy prevention medication among users 187/278 (67%). Among those with available data on measures of health indicators, about 10% had been bedridden in the past 30 days, 20.4% with WHO stage of 3/4, and about a third (32%) were eligible for ART initiation by CD4 count of ≤250 cells/mm^3^as per the RHSP criteria.

### 3.1. Prevalence and Incidence of Pregnancy during HIV Care prior to ART

Table 2 shows the prevalence and prevalence risk ratios of pregnancy among HIV+ women at enrollment into HIV care prior to ART. The prevalence of pregnancy at enrolment into HIV care was 7.2% (109/1514). Factors significantly associated with higher prevalence of pregnancy were CD4 >350 cells/mm^3^ (adj. PRR = *4.71*; 95% CI *1.41, 15.80*), being currently married (adj. PRR = 3.82; 95% CI 1.83, 7.97). Factors associated with lower pregnancy prevalence were older age 25–34 years (adj. PRR = *0.55*; 95% CI *0.34, 0.88*) or 35–45 years (adj. PRR = *0.31*; 95% CI *0.15, 0.67*) compared to women aged 15–24 years, those breastfeeding in the past 12 month (adj. PRR = *0.16*; 95% CI *0.08, 0.30*), or those using medication to prevent pregnancy (use of family planning) in the past 12 month (adj. PRR = *0.09*; 95% CI *0.02, 0.33*). 

Table 3 shows the incidence of pregnancy and incidence rate ratio of pregnancy prior to ART. The overall incidence of pregnancy during the first year in HIV care was 13.1/100 py, 95% CI (10.14, 16.75). Being currently married as compared to never married tended to have higher incidence of pregnancy, but this did not reach statistical significance, adj. IRR = 5.90 (0.87, 40.04) nor was being divorced/separated/widowed. On the other hand, the incidence of pregnancy was significantly reduced among women reporting use of medication to prevent pregnancy (nonuse of family planning) in past 12 months, adj. IRR = 0.22 (0.08, 0.61), older age (35–45 years) compared to young age (15–24 years), adj. IRR = 0.15 (0.07, 0.34), and parity of 1–3 children compared to prime gravids, adj. IRR = 0.43 (0.22, 0.86). The level of CD4 counts at entry into HIV care prior to ART initiation was not associated with incidence of pregnancy 13.2.0/100 py 95% CI (10.02,17.23) for the 351+ cells/mm^3^ compared to 12.4/100py 95% CI (5.68,23.60) for the 251–350 cells/mm^3^.

### 3.2. Prevalence and Incidence of Pregnancy during ART Use

Table 4 shows the prevalence of pregnancy and the prevalence rate ratios at the time of ART initiation. The prevalence of pregnancy was 10.1% (7.7, 12.9). The prevalence of pregnancy was higher among women with baseline CD4 of 100–250 (12.0%), compared to those with CD4 <100 (3.2%, adj. PRR = 3.24; 95% CI 1.51, 6.93). Also, the prevalence of pregnancy was significantly associated with desire for more children especially when both the woman and her spouse desired a (more) child compared to women in which both partners did not want a (more) child (adj. PRR = 2.27; 95% CI 1.04, 4.97), among currently married women (adj. PRR = 1.33; 95% CI 1.00, 1.76) compared to those not in union, and older women aged 35–45 years compared to the younger women aged 15–24 years (adj. PRR = 0.47; 95% CI 0.25, 0.91). 

Among the 566 women with data at the time of ART initiation, the overall mean (SD) CD4 counts were 159.0 cells/mm^3^ (SD = 72.7). The CD4 counts were significantly higher among the 57 pregnant women 184.0 (57.5) cells/mm^3^ compared to the 509 nonpregnant women (156.2 cells/mm^3^, *p* = 0.0013). Among the pregnant women, 24.6% (14/57) reported that only the male spouse desired to have a (another) child compared to 1.8% (1/57), where only the female did (*p* = 0.0003). 

**Figure 1:**
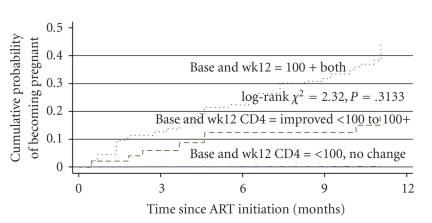
Probability of pregnancy while on ART among women, 15–45 years.

A total of 244 women were eligible for the analysis of incident pregnancy after ART initiation. The incidence of pregnancy was 24.6/100 py; 95% CI 18.1, 32.6. The incidence tended to be higher among women whose CD4 counts at the time of ART initiation were either higher than 100 cells/mm^3^ or were improved beyond 100 cells/mm^3^ by week 12 post-ART initiation (Table 5). Factors associated with high incidence of pregnancy but which did not reach statistical significance include being currently married when compared to never married (adj. IRR = 1.41; 95% CI 0.76, 2.59) and CD4 counts of 100 or higher by week 12 while on ART. Older age 35–45 years was significantly associated with lower incidence of pregnancy (adj. IRR = 0.27; 95% CI 0.15, 0.50). Pregnancy incidence tended to increase when both women and their spouses desired more children, but this increase was not significant (adj. IRR = 1.07; 95% CI 0.43, 2.63). If only the woman wanted (more) children, the incidence of pregnancy tended to be lower (adj. IRR = 0.50; 95% CI 0.09, 2.81) compared to when both partners did not want (more) children. The first viral load suppression data were available at week 24 after initiating ART. Although women with undetectable viral load had higher pregnancy rates, this was not significantly different from women with detectable viral loads, adj. IRR = 1.52; 95% CI (0.72; 3.22; results not shown in Table 5). Use of family planning methods, BMI at week 12, and WHO stage at week 12 were not associated with incident pregnancy. Figure 1 shows the Kaplan-Meier cumulative probability of incident pregnancy in the first 48 weeks while on ART treatment, by CD4 counts level at week 12. The cumulative probability of incident pregnancy increased overtime and was higher among women with CD4 count ≥100+ compared to those with CD4 counts level of less than 100 cells/mm^3^, but this difference was not statistically significant (log rank *χ* ^2^ = 2.32; *p* = 0.3133). The incidence of pregnancy prior to ART 13.1/100 py was significantly lower compared to incidence after ART initiation, 24.6/100 py (IRR = 0.54, 95% CI 0.37, 0.81, *p* < 0.0017).

## 4. Discussion

We found that the incidence of pregnancy was lower prior to initiation of ART and significantly increased while on ART. Pregnancy prevalence was significantly reduced with lower CD4 counts and tended to increase among women with viral suppression and good CD4 cell response while on ART. The incidence of pregnancy also tended to increase when both women and their spouses desired more children and decreased with older age. These findings are consistent with other studies which showed increased fertility after ART initiation [21, 22]. 

The significantly higher incidence of pregnancy after initiating ART compared to prior to ART could be due to improved immune status or reduced HIV viral load. An ethnologic study in Nigeria showed that improvements in health status after initiating ART enabled women to reassess their childbearing [24], while in another study people living with HIV (PLHIV) viewed having children as making them look forward to the future thus providing them a reason to live [1, 25]. Such views are important to be integrated in reproductive health service components of the ART programs as a way of empowering women to make appropriate reproductive health decisions. Previous studies suggest that women on HAART are significantly more likely to use contraceptives [25–27], but treatment optimism affects their fertility intentions [28–30]. In Rakai, the provision of Prevention of Mother to Child Transmission (PMTCT) interventions, including infant formula to HIV-exposed babies, might also have affected HIV+ women fertility intentions. 

Desire for (more) children, being currently married, and younger age are known to be associated with increased pregnancy. A recent study in Kenya has shown that HIV/AIDS patients have increased the desire for children and increased fertility partly due to increased infant/child mortality and reduced breastfeeding [31]. Social expectations are also associated with increased desire for more children [28], and ART is associated with higher fertility desires and intentions [28–30]. In our study, a significantly higher proportion of women reported that their spouses desired more children compared to the women. This finding was consistent with previous studies in various cultural settings [32, 33]. Men's higher desire for (more) children or their future reproductive intentions may partly be explained by their knowledge about the positive effects of PMTCT on infant health [1]. This finding suggests that reproductive health services, especially educative health messages about reproductive issues, should also be extended to involve men rather than focusing only on the needs of the female.

In summary, our data and findings from other previous studies [21, 22] show that women's fertility increases while on ART either as a result of their improved health and well-being leading to a reevaluation of their intentions and decisions regarding childbearing, or through improvement of their immunological and virological outcomes that may increase the probability of conception. Therefore, ART programs need to broaden their counseling on reproductive health services to follow the WHO (2006) *Guidelines on care, treatment and support for women living with HIV/AIDS and their children in resource-constrained settings* which recommend that the selection of ART regimen for women should consider the possibility of a planned or unintended pregnancy. 

Women in the ART programs need to be equipped with information on effective contraceptive methods to prevent pregnancy, if so desired, as well as information on the potential drug interactions with hormonal contraceptives to enable them make informed decisions on childbearing.

The other potential program/policy challenge is that pregnancy is an indicator of engagement in unprotected sex. If women and their spouses still desire to have a (another) child they will have unprotected sex, which can result in increased risk of HIV transmission to sexual partners, in addition to acquisition of multiple HIV virus strains to the already infected woman on ART. Such scenarios can lead to failure on first-line regimen further exposing the women to increased risk of morbidity and mortality. 

On the other hand, ART programs that are providing or advising women to use hormonal contraceptives should provide information on the potential risk of interaction between ARVs and some hormonal contraceptives [34] which alter the safety and effectiveness of both the hormonal contraceptives and the antiretroviral drugs. Also ART programs need to be aware of the potential risk of pill burden of both ARV drugs and contraceptives that may compromise adherence to the contraceptives or the HIV-related medication if method of contraceptive choice is pills. 

Therefore, ART programs should fully engage women and their partners whenever possible, to make them aware of such considerations while selecting contraceptive methods if they desire to use them, or the potential consequences of having unprotected sex if they still desire to have a (another) child. Full reproductive health services integration into HIV care program including family planning as well as health education should involve both men and women to help them make informed decisions on their reproductive and sexuality needs.

This study had limitations especially in the depth and scope of data that could be used to address some key issues. For example, detailed data on socioeconomic status as well as contraceptive methods and history of child bearing were not collected at the time of enrolling in HIV care. Also, the available sample size for this analysis was limited resulting in wide confidence intervals for measures of association used. However, our findings are still consistent with results from other larger studies addressing the incidence pregnancy and enrolment into ART programs.

## 5. Conclusion

In conclusion, fertility is increased among HIV-infected women after initiation of ART, and the desire by both men and women to have (more) children is still high reinforcing the need for increased reproductive health services including family planning as well as educative health messages to enable HIV-infected women and their spouses make informed decisions about their reproductive and sexuality needs.

## Figures and Tables

**Table 1:** Characteristics of women enrolling into pre-ART HIV care.

Characteristics	Number of women*	Proportion, %

Overall	1514	100
Age (years)		
15–24	199	13.1
25–34	830	54.8
35–45	485	32.0
Marital status		
Never married	155	10.2
Divorced/separated/widowed	629	41.6
Currently married	730	48.2
Sexual partner past 6 months		
None	970	64.4
One	493	32.7
2+	43	2.9

*Fertility and family planning*		
Pregnancy status		
Not pregnant	1405	92.8
Pregnant	109	7.2
Parity (ever live-births)		
Prime gravid	545	36.9
1–3	353	23.9
4+	580	39.2
Use of any medication to prevent pregnancy		
Not taking any	1236	81.6
Taking some medications	278	18.4
Medications used to prevent pregnancy		
Not taking any	1236	81.7
Injectables	187	12.4
Tablets	56	3.7
Others	34	2.3
Breastfeeding		
Not breastfeeding	1286	84.9
Yes breastfeeding	228	15.1
*General health*		
Bed-ridden in past 30 days		
No	473	89.8
Yes	54	10.3
WHO stage at initial screening		
I	476	48.3
II	309	31.3
III	151	15.3
IV	50	5.1
CD4 at initial screening		
<100	167	11.0
100–250	312	20.6
251–350	173	11.4
351+	862	56.9

Note *some totals do not add up to 1514 because of missing information on variables.

**Table 2:** Prevalence of pregnancy, unadjusted and adjusted prevalence risk ratios among HIV+ women prior to ART initiation.

Characteristics		Unadjusted	Adjusted
	Pregnant/total (%)	PRR (95% CI)	PRR (95% CI)
Overall	109/1514 (7.2)		
Age (years)			
15–24	31/199 (15.6)	1.0	1.0
25–34	61/830 (7.4)	**0.47 (0.30, 0.74)**	**0.55 (0.34, 0.88)**
35–45	17/485 (3.5)	**0.23 (0.11, 0.47)**	**0.31 (0.15, 0.67)**
Marital status			
Never married	5/155 (3.2)	1.0	1.0
Divorced/separated/widowed	15/629 (2.4)	0.74 (0.27, 2.5)	0.80 (0.28, 2.22)
Currently married	89/730 (12.2)	**3.78 (1.81, 7.90)**	**3.82 (1.83, 7.97)**
Sexual partner past 6 months			
None	63/970 (6.5)	1.0	
One	44/493 (8.9)	1.37 (0.92, 2.04)	
2+	2/43 (4.7)	0.72 (0.17, 3.07)	
Parity (ever live-births)			
Prime gravid	41/545 (7.5)	1.0	
1–3	26/353 (7.4)	0.98 (0.57, 1.67)	
4+	41/580 (7.1)	0.94 (0.64, 1.38)	
Use of any medication to prevent pregnancy			
Not taking any	106/1236 (8.6)	1.0	1.0
Taking some medications	3/278 (1.1)	**0.13 (0.03, 0.47)**	**0.09 (0.02, 0.33)**
Breastfeeding			
Not breastfeeding	103/1286 (8.0)	1.0	1.0
Yes breastfeeding	6/228 (2.6)	**0.33 (0.17, 0.64)**	**0.16 (0.08, 0.30)**
WHO stage at initial screening			
I	46/476 (9.7)	1.0	
II	14/309 (4.5)	**0.47 (0.26, 0.84)**	
III	4/151 (2.7)	**0.27 (0.13, 0.58)**	
IV	5/40 (10.0)	1.03 (0.53, 2.00)	
CD4 at initial screening			
<100	3/167 (1.8)	1.0	1.0
100–250	16/312 (5.1)	2.85 (0.89, 9.13)	2.74 (0.85, 8.82)
251–350	9/173 (5.2)	2.90 (0.95, 8.83)	2.22 (0.71, 6.98)
351+	81/862 (9.4)	**5.23 (1.54, 17.74)**	**4.71 (1.41, 15.80)**

**Table 3:** Incidence of pregnancy, unadjusted and adjusted incidence rate ratios among HIV+ women prior to ART initiation.

Characteristics	Number of women	Incident pregnancy/pyrs	Incidence/100 pyrs (95% CI)	IRR (95% CI)	Adjusted IRR (95% CI)
Overall	712	65/494.6	13.1 (10.14, 16.75)		
CD4 at screening					
251–350	130	9/72.4	12.4 (5.68, 23.60)	1.0	1.0
351+	582	56/422.1	13.2 (10.02, 17.23)	1.07 (0.57, 2.01)	0.95 (0.44, 2.05)
Age (years)					
15–24	84	14/54.5	25.7 (14.0, 43.31)	1.0	1.0
25–34	389	44/268.4	16.4 (11.91, 22.00)	0.64 (0.36, 1.12)	0.73 (0.45, 1.19)
35–45	239	7/171.7	3.5 (1.28, 7.60)	**0.16 (0.06, 0.40)**	**0.15 (0.07, 0.34)**
Marital status					
Never married	60	2/40.1	5.0 (0.60, 18.03)	1.0	1.0
Divorced/separated/widowed	310	20/217.2	9.2 (5.62, 14.22)	1.84 (0.45, 7.53)	3.83 (0.55, 26.77)
Currently married	342	43/237.2	18.1 (13.12, 24, 42)	3.62 (0.84, 15.74)	5.90 (0.87, 40.04)
Use of any medication to prevent pregnancy					
Not taking any	485	57/325.8	17.5 (13.25, 22.67)	1.0	1.0
Taking some medications	227	8/168.8	4.7 (2.05, 9.34)	**0.27 (0.10, 0.73)**	**0.22 (0.08, 0.61)**
Breastfeeding					
Not breastfeeding	572	49/397.9	12.3 (9.11, 16.28)	1.0	1.0
Yes breastfeeding	140	16/96.7	16.5(9.46, 26.87)	1.34 (0.95, 1.89)	0.43 (0.48, 1.06)
Parity (ever live-births)					
Prime gravid	301	38/211.1	18.0 (12.74, 24.71)	1.0	1.0
1–3	134	8/93.1	8.4 (3.71, 16.93)	**0.48 (0.27, 0.83)**	**0.43 (0.22, 0.86)**
4+	267	18/184.5	9.8 (5.78, 15.42)	**0.54 (0.30, 0.99)**	**0.73 (0.39, 1.36)**
WHO stage at initial screening					
I	249	20/174.5	11.5 (7.00, 17.70)	1.0	
II	133	7/90.6	7.7 (3.11, 15.92)	0.67 (0.28, 1.62)	
III/IV	35	1/22.5	4.4 (0.11, 24.76)	0.39 (0.06, 2.56)	

**Table 4:** Prevalence of pregnancy, unadjusted and adjusted PRR among HIV women at time of initiating ART.

Characteristics		Unadjusted	Adjusted
	Pregnant/total (%)	PRR (95% CI)	PRR (95% CI)
Overall	57/566(10.1)		
Baseline CD4			
<100	4/124 (3.2)	1.0	1.0
100–250	53/442 (12.0)	**3.72 (1.37, 10.08)**	**3.24 (1.51, 6.93)**
Age			
15–24	8/46 (17.4)	1.0	1.0
25–34	35/304 (11.5)	0.66 (0.34, 1.34)	0.73 (0.39, 1.34)
35–45	14/216 (6.5)	**0.37 (0.17, 0.84)**	**0.47 (0.25, 0.91)**
Desire for more children			
Both do not want	28/387 (7.2)	1.0	1.0
Only male partner wants	14/102 (13.7)	**1.90 (1.04, 3.47)**	**1.54 (0.81, 2.94)**
Only female wants	1/18 (5.6)	0.77 (0.11, 5.34)	0.78 (0.10, 5.90)
Both want	14/59 (23.7)	**3.28 (1.84, 5.86)**	**2.27 (1.04, 4.97)**
Marital status			
Not in union	22/308 (7.1)	1.0	1.0
In union	35/258 (13.6)	**1.90 (1.14,3.15)**	**1.33 (1.00, 1.76)**
HIV status disclosure			
Not to anybody	5/98 (5.1)	1.0	1.0
Yes, to somebody	52/468 (11.1)	2.18 (0.89,5.31)	1.86 (0.87, 3.99)
*FP use at ART initiation			
None	52/452 (11.5)		
Only condoms	0/8 (0)		
Other methods	0/7 (0)		

*Total less than 566 because some do not have data available.

**Table 5:** Incidence of pregnancy, unadjusted and adjusted IRR among HIV+ women after initiating ART.

Characteristics	Number of women	Incident pregnancy/pyrs	Incidence/100 pyrs (95% CI)	IRR (95% CI)	Adjusted IRR (95% CI)
Overall	244	48/195.5	24.6 (18.1, 32.6)		
Baseline and week 12 CD4					
Both <100	18	1/14.69	6.8 (0.17, 37.92)	1.0	1.0
Both 100+	182	39/145.50	26.8 (19.06, 36.64)	3.94 (0.68, 22.70)	4.12 (0.75, 22.50)
Base CD4 <100, wk12 100+	44	8/35.38	22.6 (9.76, 44.55)	3.32 (0.50, 2.26)	3.24 (0.46, 22.60)
Age (years)					
15–24	19	6/14.31	41.9 (15.39, 91.26)	1.0	1.0
25–34	133	34/103.94	32.7 (22.65, 45.71)	0.78 (0.44, 1.39)	0.82 (0.38, 1.77)
35–45	92	8/77.31	10.3 (4.47, 20.39)	**0.25 (0.13, 0.46)**	**0.27 (0.15, 0.50)**
Desire for more children					
Both do not want	179	34/144.52	23.5 (16.29, 32.88)	1.0	1.0
Only male wants	35	6/28.88	20.8 (7.62, 45.23)	0.88 (0.33, 2.33)	0.81 (0.36, 1.84)
Only female wants	8	1/6.13	16.3 (0.41, 90.89)	0.69 (0.12, 4.12)	0.50 (0.09, 2.81)
Both want	22	7/16.04	43.6 (17.55, 89.92)	*1.85 (0.84, 4.10)*	1.07 (0.43, 2.63)
Marital status					
Not married	142	23/117.13	19.6 (12.45, 29.46)	1.0	1.0
Married	102	25/78.44	31.9 (20.63, 47.05)	1.62 (0.81, 3.25)	1.41 (0.76, 2.59)
HIV status disclosure					
Not to anybody	58	15/45.69	32.8 (18.37, 54.15)	1.0	1.0
Yes, to somebody	186	33/149.88	22.0 (15.16, 30.92)	0.67 (0.38, 1.19)	0.69 (0.36, 1.32)
*Family planning method					
None	69	18/51.83	34.7 (20.58, 54.89)	1.0	
Condom only	45	12/35.45	33.8 (17.49, 59.13)	0.97 (0.47, 2.00)	
Other	11	2/8.34	24.0 (2.91, 86.67)	0.69 (0.16, 3.08)	
*BMI at week 12					
Under weight, <18.5	24	5/18.11	5 (8.96, 64.43)	1.0	
Normal weight	139	28/113.83	24.5 (16.35, 35.55)	0.89 (0.29, 2.72)	
Overweight/obese	33	5/27.23	18.4 (5.96, 42.85)	0.67 (0.14, 3.16)	
*WHO stage at week 12					
I & II	94	19/75.81	25.1 (15.09, 39.14)	1.0	
III & IV	48	6/39.27	15.3 (5.61, 33.26)	0.61 (0.23, 1.63)	

*Data not available on all the 244 women.
